# Bilateral Posterior Uveitis and Retinal Detachment During Immunotherapy: A Case Report and Literature Review

**DOI:** 10.3389/fonc.2020.549168

**Published:** 2020-11-09

**Authors:** Ling Peng, Qi-Qi Mao, Bo Jiang, Jin Zhang, Yi-Lei Zhao, Xiao-Dong Teng, Jin-Song Yang, Yang Xia, Shi-Qing Chen, Justin Stebbing, Hai Jiang

**Affiliations:** ^1^ Department of Radiotherapy, The First Affiliated Hospital, School of Medicine, Zhejiang University, Hangzhou, China; ^2^ Department of Respiratory Disease, Zhejiang Provincial People’s Hospital, Hangzhou, China; ^3^ Department of Urology, The First Affiliated Hospital, School of Medicine, Zhejiang University, Hangzhou, China; ^4^ Department of Ophthalmology, The First Affiliated Hospital, School of Medicine, Zhejiang University, Hangzhou, China; ^5^ Department of Urology, Renji Hospital, Shanghai, China; ^6^ Department of Radiology, The First Affiliated Hospital, School of Medicine, Zhejiang University, Hangzhou, China; ^7^ Department of Pathology, The First Affiliated Hospital, School of Medicine, Zhejiang University, Hangzhou, China; ^8^ Department of Respiratory and Critical Care Medicine, Second Affiliated Hospital of Zhejiang University School of Medicine, Hangzhou, China; ^9^ The Medical Department, 3D Medicines Inc., Shanghai, China; ^10^ Division of Cancer, Department of Surgery and Cancer, Imperial College London, London, United Kingdom

**Keywords:** retinal detachment, immune checkpoint inhibitor, pembrolizumab, immunotherapy, anti-angiogenesis

## Abstract

Immune checkpoint inhibitors (ICIs) cause fewer toxicities than conventional chemotherapy. Although most of the immune-related adverse events (irAEs) are mild, reversible, and manageable, potentially severe and rare irAEs remain relevant. We present a 24-year-old man with advanced hereditary renal cancer who developed bilateral posterior uveitis and retinal detachment after systematic treatment of ICI and an anti-angiogenic drug. Axitinib and pembrolizumab were administered with a partial response and following the severe ocular irAE and systemic corticosteroid treatment was initiated. Our case indicates that ocular irAEs may occur rapidly. To the best of our knowledge, this is the first case of posterior uveitis and retinal detachment in hereditary renal cancer patients treated with ICI and anti-angiogenic drugs.

## Introduction

Currently, seven ICIs have received FDA approval, targeting CTLA-4 (cytotoxic T-lymphocyte antigen 4), PD-1 (programmed death-1), or PD-L1 (programmed death ligand-1) (1–7), changing the paradigm of cancer treatment by shifting from targeting cancer itself to modulating immune cells in the tumor environment. Nivolumab, a PD-1 inhibitor, improves OS (overall survival) for patients with advanced renal cell carcinoma (RCC) following previous anti-angiogenic therapy as per data from CheckMate-025 (8). At present, pembrolizumab in combination with axitinib has become standard of care in the first line setting of advanced RCC (9, 10), which applies to all the risk groups of International Metastatic Renal Cell Carcinoma Database Consortium (IMDC) criteria. ICI blockade reverses tumor-mediated immune suppression, which expands the production of T cell immunity (11) but its use may lead to persistent T‐cell activation and unwanted side effects (12).

IrAEs due to ICIs involve different organs including skin, gastrointestinal tract, lungs, and endocrine, musculoskeletal (13). ICIs are generally well tolerated, and irAEs are generally manageable, but irAEs may sometimes lead to treatment discontinuation or withdrawal, and rarely death (14). The overall incidence of irAEs range widely with hepatic toxicities including elevated liver enzymes (35%), endocrine toxicities such as hyperthyroidism or hypothyroidism (28%), hypophysitis (7%), and pneumonitis (7%). The mechanism of irAEs is thought due to auto-reactive T and B cells escape deletion by central tolerance (15). irAEs may also be caused by epitope spreading, which contributes to cross reactivity of self and tumor antigens (16). No prospective trials have determined strategies for managing specific irAE; therefore, clinical practice is variable.

## Case Description

**Figure 1:**
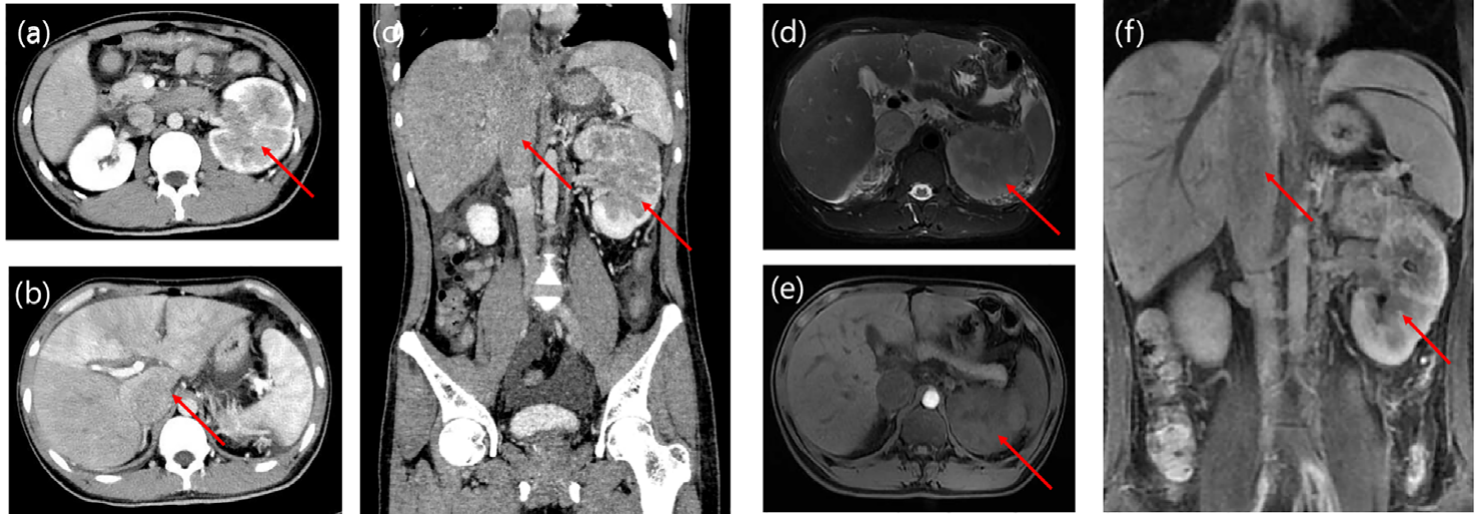
Enhanced CT **(A–C)** and MRI **(D–F)** imaging. Significant abnormal findings of left kidney and tumor thrombus of vena cava were noted *(arrow)*.

A 24-year-old male patient complained of abdominal pain in August 2018. Abdominal computed tomography (CT) and magnetic resonance imaging (MRI) scan detected a mass in his left kidney, with Mayo level IV inferior vena cava tumor thrombus (**Figure 1**). He had no family history and a radical nephrectomy and inferior vena cava thrombectomy were performed in September 2018. Intraoperative examination found the tumor extended to the right atrium and invaded the wall of the vena cava. A grayish-yellow mass with 11 cm × 7 cm in size with papillary arrangement, irregular nucleus, and large nucleoli and renal venous tumor thrombus formation was identified (**Figure 1**). Resection margins were negative, and 8 lymph nodes around the renal pedicle were negative. Histology showed was high-grade RCC, papillary type with vena cava tumor thrombus (**Supplementary Figure 1**). Immunohistology chemistry (IHC) staining indicated CK(pan)(+), p63(−), Napsin A(−), PAX-8(+), Vimentin (+), CK7(−), CD10(+), E-cadherin(+), CD117(−), P504S(+), TFE3(−). Pathological staging was pT3cN0M0, stage III.

Genomic profiling of DNA extracted from tumor specimen and peripheral white cell was performed through next-generation sequencing (NGS). NGS results revealed that TMB (tumor mutation burden) value was 4.03 mutants/Mb and the microsatellite state was stable. IHC indicated that PD-L1 Tumor Proportion Score (TPS) was 25% (**Supplementary Figure 1**).

Germline mutations of his peripheral blood revealed one *FH* (*Fumarate hydratase*) gene germline mutation p.G346Vfs*11 was identified, which has been confirmed as a pathogenic mutation. His tumor sections were stained with anti-FH and anti-2-succinocysteine (2SC) antibodies to confirm the diagnosis (**Supplementary Figure 1**). His final diagnosis was hereditary leiomyomatosis and renal cell cancer (HLRCC), an unusual and highly aggressive form of renal carcinoma. Furthermore, a *CYP2C19* p.P227P mutation was detected, which is reported to contribute to a minor (10%) pathway of axitinib metabolism (17).

Sunitinib (50-mg QD orally) was used as an adjuvant treatment after surgery. Abdominal enhanced CT in May 2019 suggested metastatic lesions in the liver and right adrenal gland (**Figure 3**). Axitinib (5-mg BID orally) was used as first-line treatment. Response evaluation by B-ultrasound after 1 month confirmed progression of disease (**Supplementary Figure 2**). Based on the results of KEYNOTE-426 which demonstrated axitinib in combination with pembrolizumab conferred a survival benefit for patients with advanced RCC in the first line setting (18), subsequent treatment with this was given from June 2019 but dosing of pembrolizumab was 100 mg every 3 weeks due to economic considerations. Response evaluation after one dose of pembrolizumab showed a partial response, with shrinkage of hepatic and right adrenal lesions, and response persisted for 5 months (**Supplementary Figure 1**). However, the patient developed vision deterioration within 3 weeks of the first dose of pembrolizumab, but he did not inform the doctor. In January 2020, his vision deteriorated quickly, and he was referred to an ophthalmologist.

**Figure 2:**
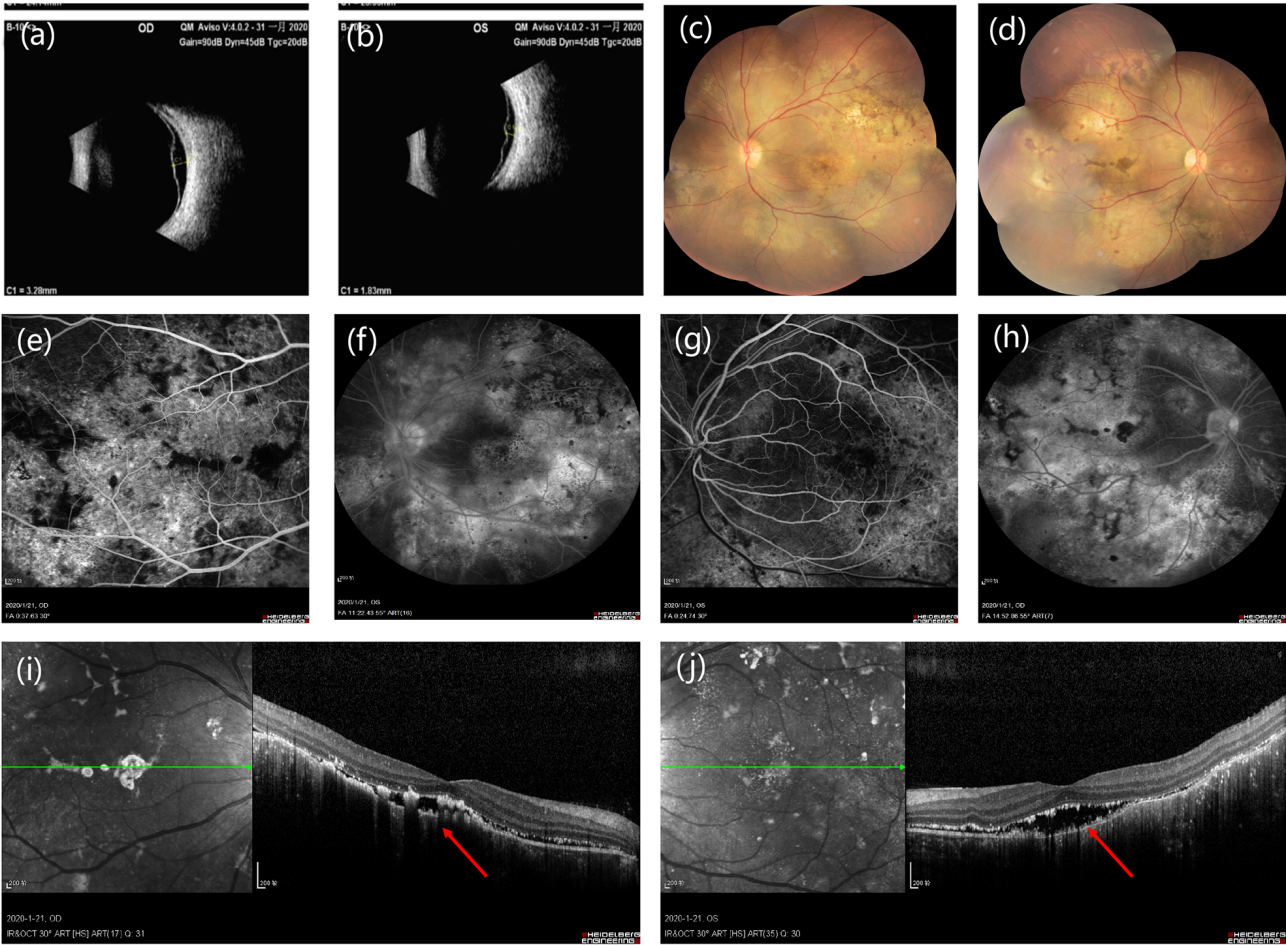
**(A, B)** Bilateral ultrasound images showing posterior retinal detachment in both eyes. **(C, D)** Fundus photographs showing large areas of depigmentation in retina. **(E–H)** Fluorescein fundus angiography (FFA) reveals early hypofluorescence of left **(E)** and right **(G)** side and late hyperfluorescence of left **(F)** and right **(H)** side in the optic disc area. **(I, J)** Optical coherence tomography of the left **(I)** and right **(J)** eye shows pockets of subretinal fluid.

A slit‐lamp examination revealed no kerato-precipitates. Tyndall effect was observed and anterior chamber (AC) cells were not obvious in both eyes. His best corrected visual acuity (BCVA) was 0.4 in the both eyes. The intraocular pressure was 13 mmHg in the right eye and 11 mmHg in the left eye. Dilated fundus examination indicated vitreous floaters were not obvious. Large areas of depigmentation were observed in retina, and local pigmentation were noticed (**Figure 2**). Posterior and inferior retinal detachment was observed. Ultrasound examination detected minimal echogenicity in the vitreous and posterior and inferior retinal detachment. Fluorescein (FFA) angiography showed dotted hyperfluorescence in early phase and a little leakage in the maculae. Optical coherence tomography (OCT) revealed neuroepithelial detachment with spotty to patchy high signal, and the choroid is thickened significantly.

**Figure 3:**
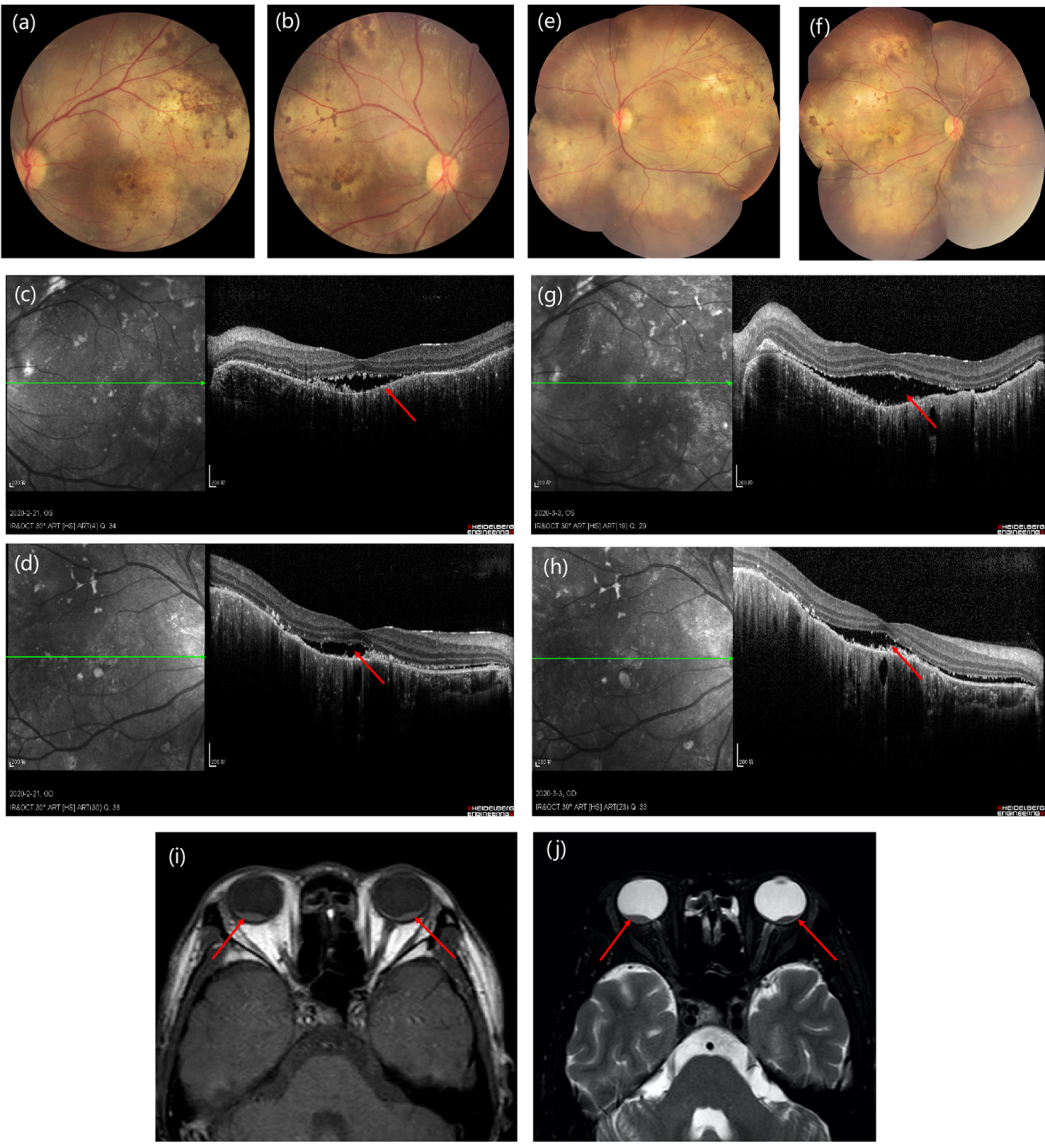
Time course of images of the presentation in fundus examination **(A, B)** and optical coherence tomography **(C, D)** at 3-week follow-up to fundus examination **(E, F)** and optical coherence tomography **(G, H)** at 6-week follow-up. **(I, J)** Eye MRI shows bilateral retinal detachment of optic nerve.

The diagnosis was posterior uveitis with bilateral retinal detachment. Immune-related tests and infection-related tests were also performed to rule out uveitis caused by infection or tumor metastasis. The complete blood count (CBC) results were normal and his erythrocyte sedimentation rate (ESR) was increased. Serologic investigations revealed negative for bacterial infection, rheumatoid factor, and tuberculosis. Serum cytokine release was tested, including IL-2, IL-4, IL-6, IL-10, TNF-α, IFN-γ, and IL-17A. Among these, IL-6 and IL-17A were increased (**Table 1**). From the time of occurrence, the ocular adverse events may due to immune checkpoint inhibitor. Based on the CTCAE (Common Terminology Criteria for Adverse Events; version 5.0), his ocular irAE was categorized as grade 3. He was treated with intravenous methylprednisolone 60 mg (1 mg/kg) systematically, followed by oral methylprednisolone. Treatment with topical prednisolone acetate (1%) every 2 h was initiated in the both eyes. Follow-ups of OCT is shown in **Figure 3**. Eye MRI at January 2020 also confirmed a thickening of posterior walls (**Figure 3**). His endocrine results indicated hypothyroidism, which was considered as an irAE, and levothyroxine tablets were commenced.

**Table 1:** Circulating cytokine level after diagnosis of posterior uveitis with bilateral retinal detachment.

Item	Result	Reference range	Unit
IL-2	3.12	0.10–4.10	pg/ml
IL-4	0.93	0.10–3.20	pg/ml
IL-6	3.21 ↑	0.10–2.90	pg/ml
IL-10	2.14	0.10–5.00	pg/ml
TNF-α	2.88	0.10–23.00	pg/ml
IFN-γ	4.53	0.10–18.00	pg/ml
IL-17A	4.46 ↑	0.10–2.90	pg/ml
CRP	0.6	0–8.0	mg/L
ESR	23	0–15.0	mm/hour

IL, interleukin; TNF, tumor necrosis factor; IFN, interferon; CRP, C-reactive protein; ESR, erythrocyte sedimentation rate.

Our multidisciplinary team decided to permanently discontinue his use of pembrolizumab. At follow-up in February 2020, progressive disease in his adrenal, hepatic and bones were observed (**Supplementary Figure 3**). There was no indication of metastatic lesions in his eyes. Third-line systemic therapy of bevacizumab and erlotinib was initiated, and the patient responded well to the combination therapy. The timeline of his diagnosis, treatment and onset of irAE were shown in **Supplementary Figure 4**.

## Discussion

Ocular adverse events are rare, occurring in less than 1% of patients treated with ICIs (19). A systematic review and meta-analysis of ocular toxicities due to ICIs reported that the most common ocular toxicities included uveitis and dry eyes. Pooled analysis for OR (odds ratio) of all-grade immune-related ocular toxicities is 3.40 (95% CI: 1.32–8.71) (20). Due to the inherent characteristics of cohort studies, the prevalence of rare AEs such as ocular toxicities is somewhat underestimated, so the real incidence might be higher than expected (21). A disproportionality analysis of U.S. Food and Drug Administration (FDA) database FAERS demonstrated the reported odds ratios (RORs) of ICIs. Among approved ICIs, nivolumab ranks the highest of ocular adverse events associated with ICI (ROR = 10.54, 95% CI 7.30 - 15.22) (22).

The precise causal relationship in the scenario of combination therapy is difficult to make a decisive conclusion. As previously reported, targeted therapy and other anti-cancer drugs may also cause ocular toxicities (23). The association of oral anti-VEGF drugs with retinal detachments, while rare, is plausible. But the association of oral anti-VEGF drugs with post uveitis and retinal detachment are unclassified although this suggests a “signal” requiring further study (24). Currently, various anti-angiogenic drugs, such as anti-VEGF antibody (ranibizumab), VEGF trap (aflibercept), and multikinase tyrosine kinase inhibitor (axitinib) are currently used to treat corneal neovascularization. Sunitinib malate at the concentration of 12.5 mg/ml caused no toxicity, but 25 mg/ml caused retinal changes suggesting toxicity in the *in vivo* model (25). There is only an observational case report of impaired retinal circulation of axitinib in treating patient with RCC (26).

We searched FAERS database of spontaneous reports of uveitis and retinal detachment of ICIs using web search tool (https://www.pharmapendium.com/home), using the generic drug name “atezolizumab, avelumab, cemiplimab, durvalumab, ipilimumab, nivolumab, and pembrolizumab”, AND “uveitis”, AND “retinal detachment” up to February 20, 2020. A total of 184 uveitis following ICIs were reported in the database, among which eight cases with uveitis and retinal detachment were identified, including five patients who received nivolumab and three patients with pembrolizumab. We also searched reported cases with sunitinib and axitinib, and there were no cases of uveitis with retinal detachment. The onset time of vision change occurs 3 weeks after initiation of pembrolizumab of this patient, therefore, we suspected the ocular toxicity of this patient were primarily due to pembrolizumab. A retrospective study reported the average onset time of ocular immune-related adverse events (irAEs) from initiation of ICI measured 15.7 weeks (27), but cases also reported ocular irAE onset within 20 days of ICI use (28, 29).

We also searched published literature regarding ICI-related retinal detachment, identifying nine cases with retinal detachment in patient receiving ICIs (**Table 2**) (30–38). Among them, most of the cases reported were anti-CTLA-4 antibodies. Anti-CTLA-4 agents have a higher function by enhancing T-cell priming, while blockade of PD-1/L1 pathway resulting in re-invigorating CD8 T-cell responses (39). IrAEs are more common in patients treated with CTLA-4 blockades than PD-1/L1 inhibitors, reflecting their different roles in immune regulation (40).

**Table 2:** Cases of retinal detachment following ICI: clinical characteristics and malignancy status.

Patient No.	Reference	Sex	Age	Malignancy	ICI	Onset	Cancer status	ICI discontinuation	Type of RD
1	Current paper	M	24	HLRCC	Pembrolizumab	3 weeks	PR	Yes	ERD
2	Miyakubo (30)	M	78	Melanoma	Ipilimumab	15 weeks	NR	No	SRD
3	Wang (31)	F	64	RCC	Nivolumab	12 weeks	NR	No	SRD
4	Rapisuwon (32)	F	60	Melanoma (Uveal)	Ipilimumab + Nivolumab	4 weeks	Near CR	Continue on nivolumab	SRD
5	Obata (33)	F	63	Melanoma	Nivolumab	24 days	NR	No	SRD
6	Telfah (34)	M	58	Melanoma	Pembrolizumab	52 weeks	PR	Yes	ERD
7	Tsui (35)	M	60	Melanoma	Ipilimumab+ Nivolumab	5 weeks	NR	No	ERD
8	Theillac (36)	M	55	Melanoma	Nivolumab	4 weeks	NR	Yes	SRD
9	Crews (37)	M	46	Melanoma	Ipilimumab	6 weeks	NR	Yes	SRD
10	Mantopoulos (38)	F	70	Melanoma	Ipilimumab	28 weeks	CR	Yes	SRD

NR, not reported; CR, complete remission; PR, partial remission; RD, retinal detachment; SRD, serous retinal detachment; ERD, exudative retinal detachment; M, male; F, female.

Timely detection of ocular symptoms and subsequent treatment are vital to the outcome of irAEs. Most of the symptoms are not typical, and are easily confounded by underlying co-morbidities, so mild manifestations of ocular toxicities are commonly overlooked. For example, visual deterioration may have different etiologies such as myopia and cataract, etc. Differentiating ocular irAE from other causes has important implications for optimal treatment, including infection, tumor metastasis to eye, and other possible reasons. Patients complaining of eye symptoms while receiving ICI should be seen by an ophthalmologist (21). Therefore, as the occurrence of ocular toxicities, other causes must always be ruled out. A high level of suspicion is needed to monitor and treat ocular toxicities, while in real-world setting, great variation exists. Apart from hypothyroidism and few other possible etiologies during ICI treatment, agreement among observers was poor (41).

In order to rule out the possibility of cancer-related retinopathy, infection and tumor metastasis, observation of ocular findings using multiple imaging analysis can provide insights into possible etiology of the condition. Whole-body PET/CT can be useful for clinical evaluation of patients with uveal metastases (42). The eyes are considered as an immune privileged site, but ocular irAEs can involve any component of the ocular apparatus (43). Patients experiencing ocular adverse events (AEs) may present with any of the following symptoms: blurred/distorted vision, blind spots, change in color vision, photophobia, tenderness/pain, eyelid swelling, and proptosis. The most common adverse effects include dry eyes, conjunctivitis, uveitis, and myasthenia gravis (19). Referring to an ophthalmologist is suggested if the symptoms occur. It is recommended that patients should receive ocular examination including visual acuity in each eye, color vision, pupil size, shape, and reactivity, red reflex, and fundoscopic examination (44). During evaluation of vision, sight-threatening effects are ocular myasthenia, corneal punctate epithelial erosions, subconjunctival hemorrhage, corneal perforation, uveitis, hypotony maculopathy, cystoid macular edema, and serous retinal detachment (45). Here, the irAE involves bilateral posterior and retina. Certain parts of the eye are unable to regenerate after destructive inflammation, such as neural retina (46).

There are several guidelines regarding the management of irAEs, including those published by ESMO, SITC, and ASCO/NCCN (47–49). However, the treatment of rare irAEs such as ocular toxicity is not fully clear due to lack of adequate evidence (50). NCCN guidelines recommend the use of systemic corticosteroids and tropical steroids as initial treatment (47). If the ocular irAE is grade 1~2, it is possible to continue prior ICI balancing the risk/benefit ratio for each individual patient. The time to resolution of irAEs depends on different organs involved (51). In our case report, this patient received steroids for more than a month, while his vision remained stable. In response to the acute inflammatory phase, many cytokines are continuously secreted, notably IL (interleukin)-1, IL-6, and human tumor necrosis factor α (TNFα). An assessment of baseline cytokine level assessment before ICI therapy followed by repeated measurements in case of irAE might be useful.

There are some other issues that require addressing. Firstly, the irAEs in different underlying malignancies may also vary. A systemic review compared the likelihood of irAEs in different tumor types, including RCC, melanoma, and non-small cell carcinoma (NSCLC) (52). HLRCC is a type of familial renal cancers which is driven by *FH* gene mutation, which is the most aggressive tumor among the familial renal cancer syndromes (53). Most of HLRCCs do not express PD-L1 (54), while in our case the expression of PD-L1 of tumor cell was 25%. There were not enough evidences to support whether this special subtype of RCC would have any link to this rare irAE.

Secondly, combinations of ICI with other drugs such as ICI, chemotherapy or anti-angiogenic drug may lead to higher incidences of irAEs (55, 56). Combination therapy with PD-1 inhibitors and chemotherapy have similar side effects to those of chemotherapy alone; however, combination therapy with CTLA-4 and PD-1 inhibitors may lead to more severe AEs than those with monotherapy (57).

Thirdly, the addition of ICI on the basis of axitinib has led to shrinkage of his hepatic and right adrenal metastatic lesions, while he developed serious ocular toxicities. Literature suggests irAE onset is predictive of anti-PD-(L)1 antibody response across a variety of solid tumors (58–60). However, the relationship of efficacy and toxicity in the ICI evokes debate regarding the “survivor bias” phenomenon (61).

In conclusion, this is the first report of posterior uveitis and retinal detachment with severe vision deterioration in a HLRCC patient receiving ICI and anti-angiogenic drug. The use of ICIs in medical oncology continues to rise significantly; therefore, the number of patients with rare irAEs will increase. There is a need for routinely querying patients during their treatment regarding the occurrence of unexpected adverse events. This case highlights the monitoring and timely detection of rare irAEs in RCC patients receiving ICIs.

## Ethics Statement

The patient provided his written informed consent for the publication of images or data included in this article.

## Author Contributions

All authors listed have made a substantial, direct, and intellectual contribution to the work, and approved it for publication.

## Funding

This study was partially supported by Natural Science Foundation of Zhejiang Province, China (Grant number: LY19H160041).

## Conflict of Interest

SQC was employed by the company 3D Medicines Inc. JS is editor-in-chief of Oncogene. JS has sat on a number of scientific advisory boards, including Benevolent AI, and consults with Lansdowne partners and Vitruvian; he sits on the Board of Directors for BB Biotech Healthcare Trust and chairs Xerion Healthcare.

The remaining authors declare that the research was conducted in the absence of any commercial or financial relationships that could be construed as a potential conflict of interest.

## Supplementary Material

Supplementary Figure 1 Histological and genetic assessment of tumor. **(a)** Histopathologic findings of renal papillary carcinoma (Hematoxylin and Eosin, 100×). Large atypical cells with marked nucleus proliferate diffusely. **(b)** PD-L1 staining of the specimen (100×). **(c)** IHC confirmed loss of staining for FH (100×). **(d)** Positive staining of 2-succinocysteine (100×). **(e)** TMB value was 4.03 mutants/Mb. **(f)** Microsatellite state was stable.

Supplementary Figure 2 Radiological mages of tumor evaluation. **(a)** Hepatic metastatic lesion of CT sca*n* on May 2019 (arrow). **(b)** Right adrenal metastatic lesion of CT scan on May 2019 (arrow). **(c, d)** Ultrasound confirmed hepatic and right adrenal lesions on June 2019 (arrow). **(e, f)** Abdominal MRI indicated shrinkage of hepatic lesion and right adrenal metastasis (arrow) on July 2019. **(g, h)** Abdominal MRI indicated remission of right adrenal metastasis on November 2019 (arrow).

Supplementary Figure 3 A positron emission tomography/computed tomography scan identified increased fluorodeoxyglucose uptake in the **(a)** liver, **(b)** right adrenal gland, **(c, d**) nerve roots and **(e, f)** vertebrae (arrow).

Supplementary Figure 4 Clinical course of the patient.
