# Supplementary figures and images for: Bilateral Posterior Uveitis and Retinal Detachment During Immunotherapy: A Case Report and Literature Review
Source: Front Oncol. 2020 Nov 9;10:549168. doi: 10.3389/fonc.2020.549168 (PMC7680916; doi:10.3389/fonc.2020.549168)

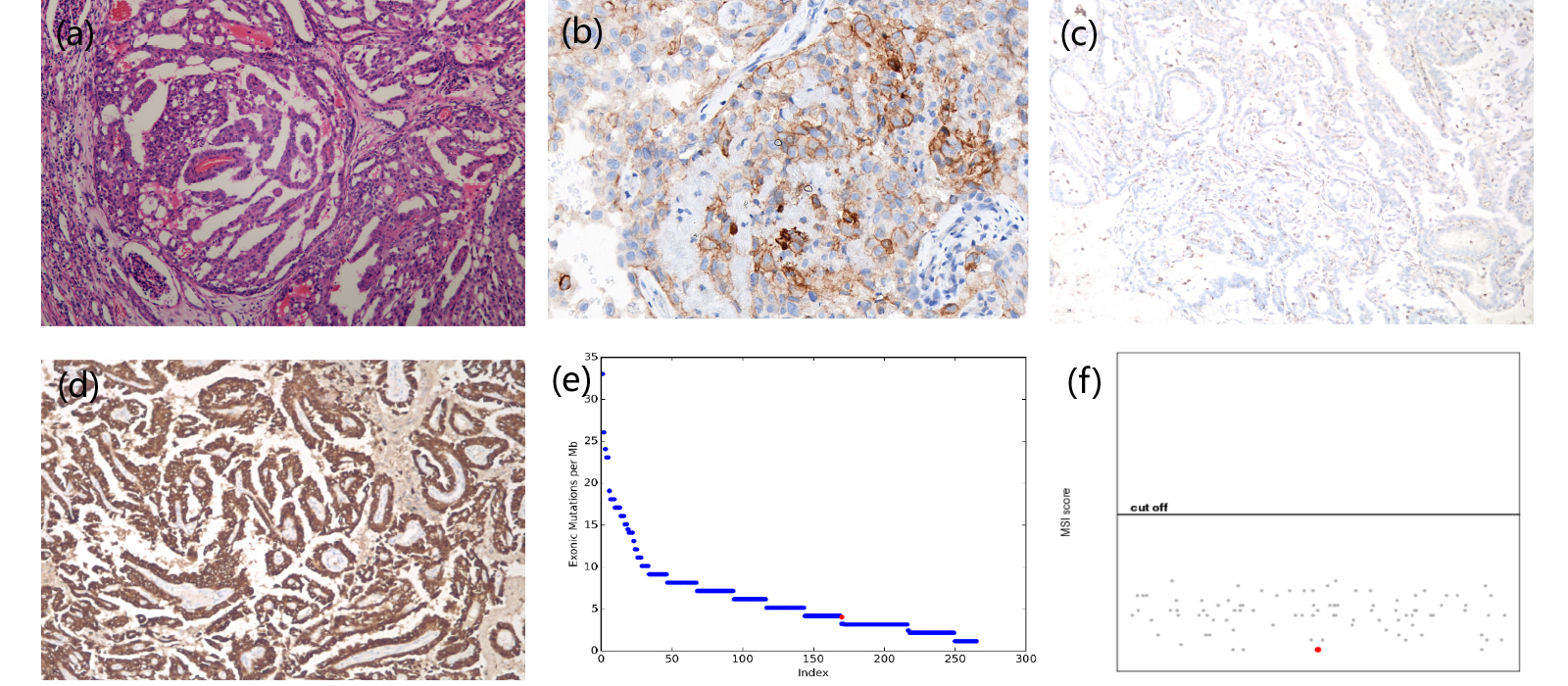

Supplement: Supplementary Figure 1 — Histological and genetic assessment of tumor. (a) Histopathologic findings of renal papillary carcinoma (Hematoxylin and Eosin, 100×). Large atypical cells with marked nucleus proliferate diffusely. (b) PD-L1 staining of the specimen (100×). (c) IHC confirmed loss of staining for FH (100×). (d) Positive staining of 2-succinocysteine (100×). (e) TMB value was 4.03 mutants/Mb. (f) Microsatellite state was stable. [file Image_1.jpeg]

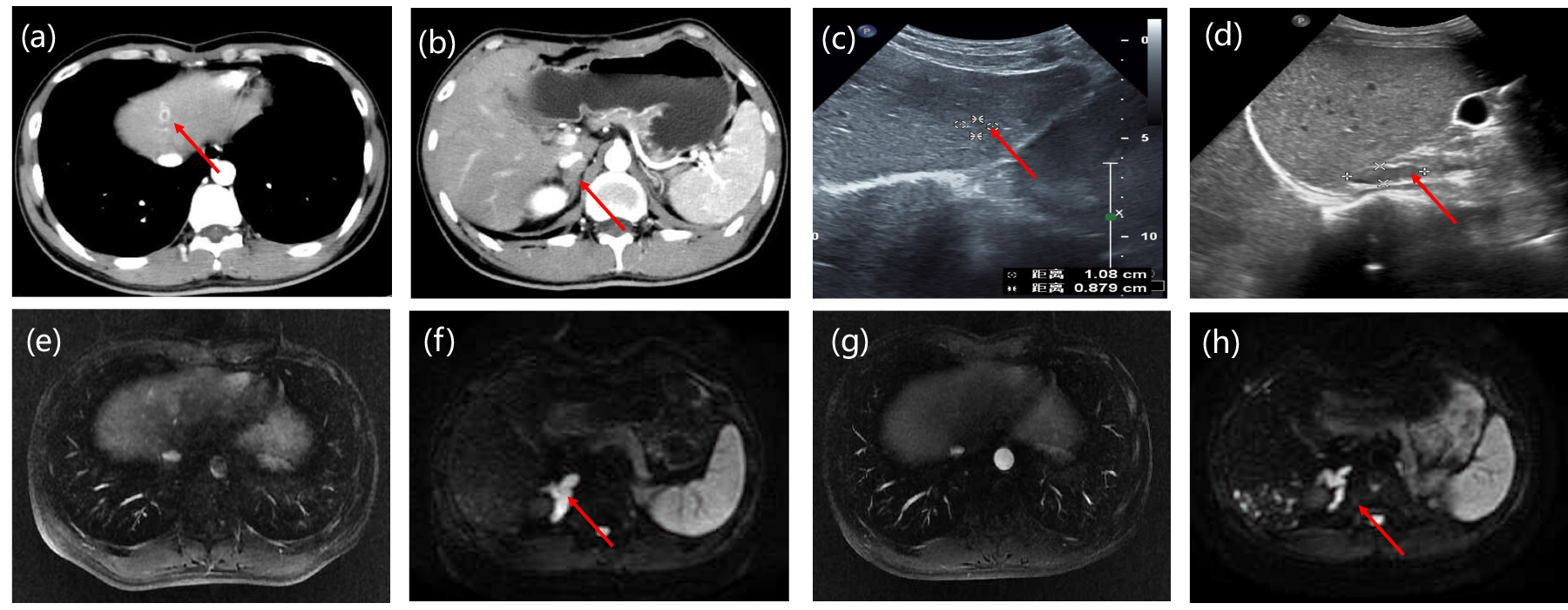

Supplement: Supplementary Figure 2 — Radiological mages of tumor evaluation. (a) Hepatic metastatic lesion of CT scan on May 2019 (arrow). (b) Right adrenal metastatic lesion of CT scan on May 2019 (arrow). (c, d) Ultrasound confirmed hepatic and right adrenal lesions on June 2019 (arrow). (e, f) Abdominal MRI indicated shrinkage of hepatic lesion and right adrenal metastasis (arrow) on July 2019. (g, h) Abdominal MRI indicated remission of right adrenal metastasis on November 2019 (arrow). [file Image_2.jpeg]

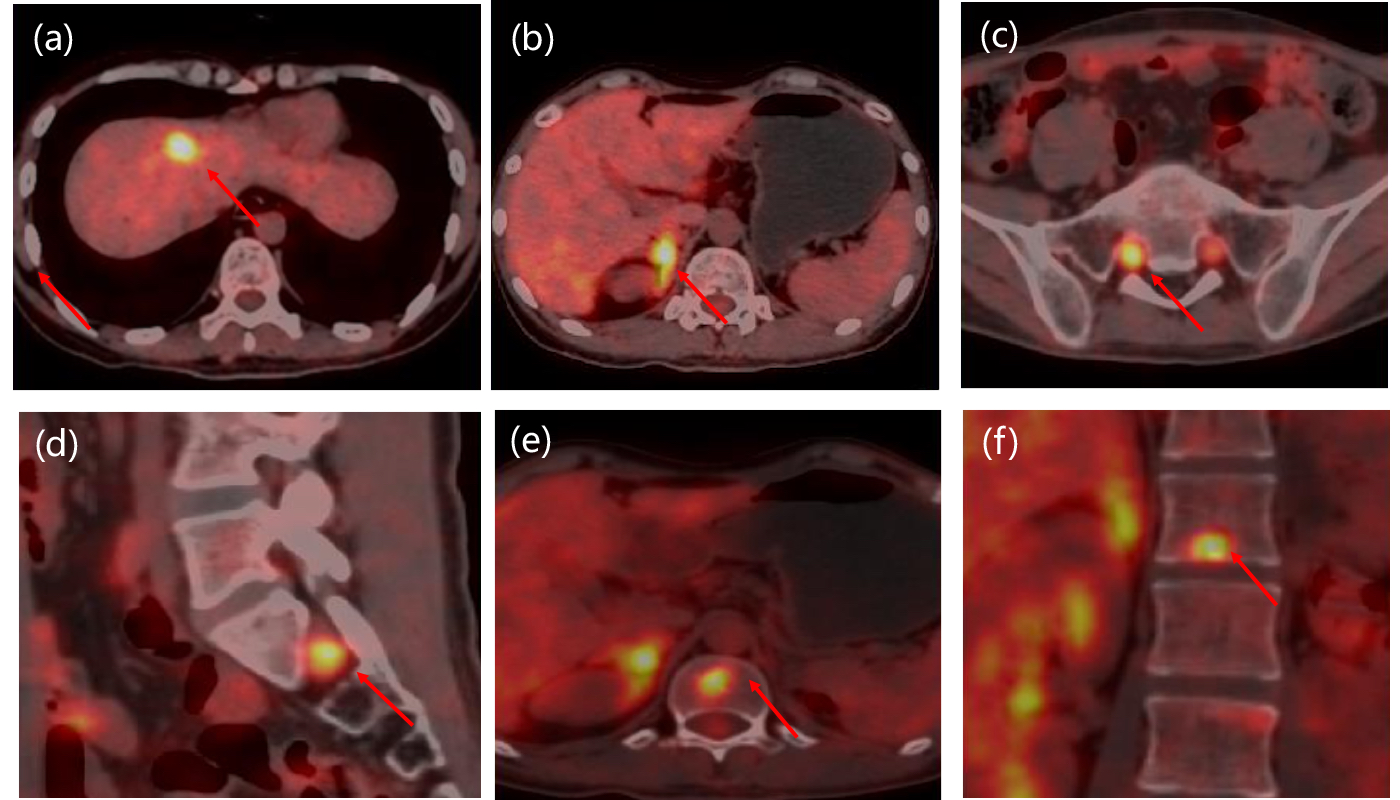

Supplement: Supplementary Figure 3 — A positron emission tomography/computed tomography scan identified increased fluorodeoxyglucose uptake in the (a) liver, (b) right adrenal gland, (c, d) nerve roots and (e, f) vertebrae (arrow). [file Image_3.jpeg]

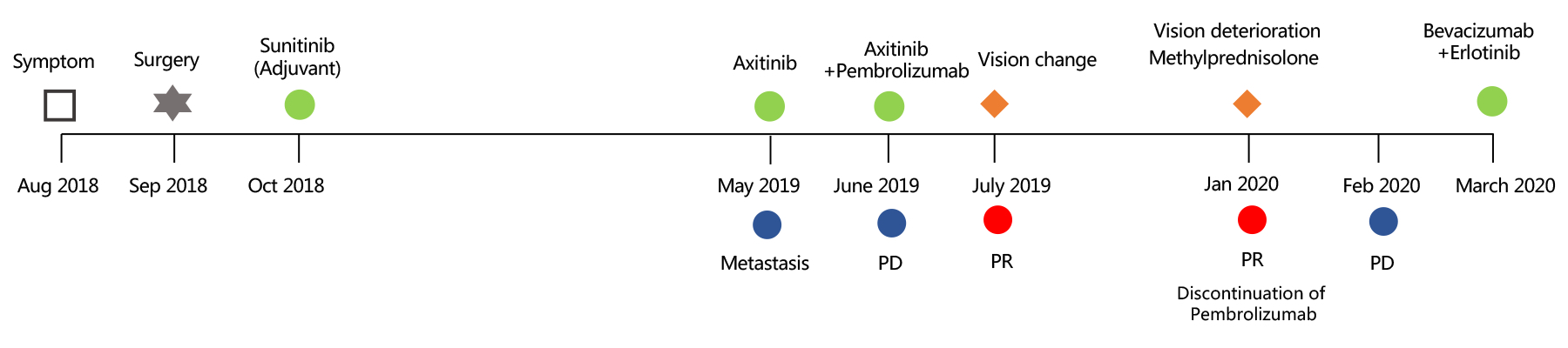

Supplement: Supplementary Figure 4 — Clinical course of the patient. [file Image_4.jpeg]
